# Conscious experience and emotion: an attention-based account

**DOI:** 10.3389/fpsyg.2026.1738304

**Published:** 2026-02-02

**Authors:** Giorgio Marchetti

**Affiliations:** www.mind-consciousness-language.com, Setteville BL, Italy

**Keywords:** affect, attention, emotion, energy, set-point, the AME theory of consciousness

## Abstract

This article aims to explain how emotions arise and operate within the framework of my attention-based theory of consciousness, referred to here as the AME theory of consciousness (Attentional Modulation of Energy). According to the AME theory of consciousness, the phenomenal aspect of consciousness is produced by the modulation of the energy level of the area of the organ of attention (aOA) that underpins our attentional activity. The phenomenal aspect of consciousness, in turn, provides us with a sense of self and informs us about how our activities affect it. It manifests through five main dimensions—qualitative, quantitative, hedonic, temporal, and spatial—each of which can be explained by a specific aspect of the modulation of the energy level of the aOA. Emotions, which represent some of the most informative forms of conscious experience, emerge from the interaction of three main components—core affect, cognitive appraisal processes, and physiological and behavioral manifestations—whose interplay unfolds through cycles of conscious and unconscious processing. They arise when an object elicits an affective response capable of shifting the focus of attention from the object to the sense of self. This shift results in the activation of an aOA related to the sense of self (or to an aspect of it) and leads to the adoption of a corresponding set-point. Deviations from this set-point generate the conscious experience of emotion, which informs the individual about the state of his internal equilibrium and the integrity of his sense of self. Emotions thus act not only as adaptive regulators of behavior but also as fundamental operations through which the individual monitors, defines, and continually reconstructs his sense of self.

## 1 Introduction

Conscious experience—whether it manifests as a sensation, a feeling, an emotion, a perception, an idea, a memory, an utterance, or in another form—is the primary and most fertile means we have of becoming informed about how objects in the world (I use the terms “object” and “objects” for the sake of simplicity, in a very general sense, to refer not only to physical objects but also to imagined objects, living beings, events, ideas, or other things) relate to us: what kinds of effects they have on us, how they limit us, how important they are for us, whether and how they help satisfy our needs, how our activity can or cannot modify them, how we can exploit their affordances, and so on.

By informing us about how objects relate to us, consciousness provides us with two important kinds of knowledge. On the one hand, it allows us to understand what significance objects have for us and to assign meaning to them. In this view, consciousness can be defined as the organ of meaning (Marchetti, 2010). On the other hand, it helps define the boundaries of our sense of self—who we are, what we are able to do and what we are not, how limited we are in our movements, what we can control and what we cannot, what belongs to us and what does not, how our perspective differs from that of others, and so on. Every conscious experience continuously (re)defines, sustains, and helps maintain each human being’s unique individual identity (Marchetti, 2024). Indeed, our sense of self is not a fixed entity existing independently, but rather a dynamic one (Fingelkurts et al., 2020; Davey and Harrison, 2022) that develops over time (Rochat, 2003; Cleeremans, 2008)—already *in utero* (Ciaunica et al., 2021)—through the continuous experiences we undergo with other objects (moreover, our sense of self also modifies every time we intentionally reflect on our own capacities).

Particularly relevant to the effectiveness, comprehensiveness, and overall usefulness of the knowledge provided by conscious experience is its affective dimension. The affective dimension gives us an immediate appraisal of objects by informing us about the subjective valence they have for us—that is, whether they affect us positively or negatively, whether they are attractive or repulsive, arousing or calming, pleasant or unpleasant. In this way, the affective dimension enables us to distinguish hostile from hospitable objects and to organize adaptive responses to them (Cacioppo et al., 2012).

The affective dimension is usually described as consisting of an integral blend of hedonic (pleasure–displeasure) and arousal values (Russell, 2003)—even though additional factors have been identified, such as dominance, or the degree of control one feels in relation to an object (see, for example, Russell and Mehrabian, 1977)—and is considered central to phenomena such as emotions, moods, and sentiments (Russell, 2003; Scherer, 2005; Carver and Scheier, 2012).

Although the term *affect* is often used as an umbrella term to refer to this restricted set of phenomena (Bagozzi et al., 1999; Scherer, 2005; Naven, 2013), it should be noted that the affective dimension characterizes and constitutes all forms of conscious experience (Cabanac, 1996, 2002; Russell, 2003; Solms, 2019; Marchetti, 2022). As Russell (2003, p. 149) points out:

“Objects and events all have affective qualities, the perception of which is as much a part of contact with the external world as perception of nonaffective qualities. Indeed, just as sensation, perception, and cognition cannot be cleanly distinguished from one other, perception of affective quality is another part of this processing of information.”

Among the various types of conscious experiences, emotions are those which, by leveraging most on the affective dimension, prove to be the most informative. Emotions offer an immediate, synthetic evaluation—based on a complex, multidimensional appraisal—of the relevance that something currently has for our wellbeing, goals, or values. For example, fear signals that something is threatening me now; joy signals that something is supporting me now; and anger signals that something is currently violating what I believe is right. At the same time, emotions also guide our behavior by indicating appropriate actions—both mental and physical—for dealing with or adapting to the object that has elicited them. Moreover, emotions often—though not always—prompt us to categorize and communicate the emotional episode to others (an extensive and quite comprehensive account of the processes involved in emotions can be found in Scherer’s Component Process Model: Scherer, 2005, 2009; Grandjean et al., 2008; Scherer and Moors, 2019).

The high informativeness of emotions distinguishes them from other forms of conscious experience, whose roles may range from a purely or predominantly informative one—such as sensations and perceptions, which provide real-time data about the internal and external environment—to that of generating internal drives for action, as in the case of motivations and intentions, or that of enabling the organism to anticipate and evaluate potential actions and scenarios without needing to enact them physically, as with imagination and mental simulations.

The main aim of this article is to provide an explanation, within the framework of my theory of consciousness (Marchetti, 2018, 2022, 2024)—referred to here as the AME theory of consciousness (Attentional Modulation of Energy)—of how the complex informational content of emotion is generated and how it contributes to the definition and maintenance of the sense of self. To this end, I will first introduce the AME theory of consciousness and its account of the sense of self, and then describe the main components of emotion.

Before proceeding, however, some clarifications are in order regarding whether emotions necessarily have an object, and whether unconscious emotions can exist.

While the majority of scholars argue that emotions have an object (Sander, 2013), some argue—on various grounds—that emotions do not necessarily require one (see, for example, Lamb, 1987; Damasio, 1999; Shargel, 2015). Examples often given of objectless emotions include euphoria, depression, and apathy, and what Damasio calls background emotions, such as wellbeing, and tension. In my opinion, this controversy stems primarily from a terminological issue. Much depends on how the term “object” is defined. Defining “object” as something external to the experiencing subject leads to accepting the existence of objectless emotions, whereas including also sensations from within the subject (e.g., proprioceptive and interoceptive sensations) in the definition of “object” (as I do) leads to rejecting the existence of objectless emotions. Indeed, emotions such as euphoria, depression, and apathy, as well as background emotions like wellbeing and tension, are usually intransitively experienced and perceived with reference to oneself. An additional source of confusion is how the term “emotion” is defined. Some authors prefer to categorize affective states that do not have an external object—such as euphoria, anxiety, wellbeing, and tension—as “moods” rather than “emotions,” thus avoiding the need to address the possibility of objectless emotions. In a similar vein, it is no coincidence that Damasio (1999) felt the need to introduce the category of “background emotion” alongside the canonical primary and secondary ones: “I will talk about three levels of emotion—background, primary, and secondary. This is revolutionary enough for one day, given that background emotions are not part of the usual roster of emotions” (Damasio, 1999, p. 341). Finally, it should be noted that the argument about the existence of objectless emotions can, at least in some cases, be rebutted on the grounds that emotions may sometimes appear objectless because their object is inexpressible, not properly formulable, or even unconscious.

The second clarification concerns the existence of unconscious emotions. In this article, I deal specifically with conscious emotions. By qualifying emotion as a conscious phenomenon, I do not intend to deny that there may be cases in which emotions are not felt, nor do I deny that certain components of emotion (e.g., emotion-eliciting stimuli, autonomic changes, motor expressions) may go unnoticed. On the contrary, I simply mean to prioritize the conscious aspect over the unconscious one when scientifically addressing emotions. This is because conscious experience is the privileged form of knowledge we have of them. After all, if we can talk about emotions and if emotions can be studied scientifically, it is only because we have some conscious experience of them or their effects. Without any conscious experience of emotions or their effects, we would neither know of their existence, nor would scientists know what they need to explain and investigate in the first place when dealing with emotions (Barrett et al., 2007).

The topic of the existence of unconscious emotions has not been without controversy. On the one hand, some scholars argue clearly that it does not make much sense to speak of unconscious emotions (Clore, 1994; LeDoux, 1994; LeDoux and Brown, 2017). For example, LeDoux (1994, p. 291) maintains: “Emotions are affectively charged, subjectively experienced states of awareness. Emotions, in other words, are conscious states. While nonconscious emotions do not exist, conscious emotional states are produced by unconscious processes.” On the other hand, other scholars (Öhman et al., 2000; Berridge and Winkielman, 2003; Winkielman and Berridge, 2004; Prinz, 2005; Winkielman et al., 2005; Winkielman and Hofree, 2012), based on empirical evidence and conceptual considerations, argue that emotions are not always conscious. As Winkielman and Hofree (2012, p. 3366) specify, “The absence of consciousness can come in the form of (1) unawareness of the stimulus eliciting the emotion or (2) unawareness of the emotion itself, producing an emotion that is not subjectively felt.” For example, Winkielman et al. (2005) showed that subliminally presented emotional faces can cause affective reactions that alter consumption behavior, without eliciting conscious feelings when the affective reactions are caused. In their experiments, the participants were first subliminally exposed to happy, neutral, or angry faces embedded in a cognitive task requiring them to classify a clearly visible neutral face as male or female. They were then asked to rate the pleasantness of a fruit beverage. The experiments showed that those who had been shown happy faces not only rated the pleasantness of the beverage higher than those shown angry or neutral faces, but also consumed larger amounts of the beverage than those shown angry or neutral faces. Importantly, the participants reported no differences in how they felt emotionally. Therefore, the participants’ behavior indicated that emotions influenced their actions, even though the emotions were not consciously detected.

Some scholars have addressed this controversy by excluding conscious experience from their definition of emotion. Damasio (1999, 2010), for example, reserves the term feeling to describe what others might call conscious emotion. For him, emotions are unconscious processes involving lower, more automatic parts of the brain (e.g., the brainstem, amygdala, and hypothalamus) and the body. Emotions become conscious as feelings when higher brain areas (such as the cerebral cortex) recognize and interpret the bodily changes caused by emotions. Likewise, for Prinz (2005, p. 9), “emotions are feelings when conscious, and they are not feelings when unconscious.”

Apart from this terminological solution, a more promising way out of the controversy over the conscious nature of emotions can be derived from the notion of levels of processing (see, for example, Grandjean et al., 2008; Sander and Delplanque, 2021). In its essence, this notion holds that emotional information can be processed at varying depths or extents: information that is only superficially or minimally processed remains unconscious, while deeply or extensively processed information becomes conscious and can eventually be verbalized. If we adapt this notion to the AME theory of consciousness, we find that the controversy regarding the conscious nature of emotions is resolved. As we will see, according to the AME theory of consciousness, the most important process that determines whether information is consciously experienced is attention: information becomes conscious when, and to the extent that, it is processed by attention. All other conditions being equal, if attention fully processes information, information becomes conscious; if it only partially processes information, that is, below a certain threshold, information may remain partly unconscious; and if it does not process information, information remains unconscious. If we apply this adapted version of the notion of levels of processing—which we may term “levels of attentional processing”—to emotions, we can readily explain various cases of emotional unawareness or unconscious emotions.

Firstly, consider the case of complete emotional unawareness described by Prinz (2005). You are lying in bed when the sound of a window breaking in another room startles you. You instantly assume it might be a burglar and concentrate intensely on the noise, trying to detect any further sounds. Although your body immediately reacts with a fear response, you do not consciously feel the fear because your attention is fully absorbed in listening for any signs of an intruder. After a moment of silence, you hear your cat scrambling around, and it dawns on you that she must have knocked something over. Only then do you notice that your heart is racing, your breathing is strained, and your body is tense with fear. You were afraid all along, but you did not realize it because your focus was entirely on listening for danger.

Secondly, consider the case of unawareness of the emotion demonstrated by Winkielman et al.’s (2005) experiments. The participants’ failure to report any change in emotion, despite the fact that emotions do alter their behavior, can be accounted for by differences in the attentional processing involved in feeling an emotion versus registering, reflecting on, and reporting the felt emotion. In these cases, the participants would actually experience a certain emotion but would be unable to consciously reflect on and report it (Whiting, 2018).

Thirdly, the notion of levels of attentional processing can also readily accommodate evidence showing that individuals are not always aware of all autonomic changes (e.g., pupil dilation or electrodermal response), motor expressions, or action tendencies induced by an emotional stimulus, nor of the source that elicits the emotion (see, for example, Öhman and Soares, 1994; LeDoux, 1996; Moors, 2010; Morsella and Bargh, 2011). When these elements are not processed by attention, they remain unnoticed.

## 2 The main dimensions of consciousness

Consciousness is the fundamental means by which we gain knowledge of objects and of our self. Whatever type of conscious experience we have—whether a sensation, perception, feeling, emotion, mood, memory, idea, utterance, dream, hallucination, or something else—it informs us of how objects relate to us, the significance they hold for us, and the boundaries, needs, abilities, and other attributes of our self.

The knowledge provided by consciousness inevitably takes on the features of the specific conscious experience from which it arises. The knowledge we gain of a person in direct, face-to-face interaction differs from that acquired through indirect means, such as hearsay or reading about him or her in the newspapers: whereas the former is characterized by vivid sensory and perceptual qualities (the dimensions of our interlocutor’s body, the shape and color of his or her eyes and hair, the sound of the voice, the way he or she moves and walks, etc.), the latter lacks such immediacy and is instead shaped primarily by our imaginative abilities.

These features, shaped by the particular conscious experience through which knowledge emerges, result from the modulation and interplay of the main dimensions of consciousness.

Across the various types of conscious experience, five main dimensions of consciousness can be identified: *qualitative, quantitative, hedonic, temporal, and spatial* (Marchetti, 2022, 2024). The qualitative dimension corresponds to the *what-it-feels-like* (Nagel, 1974) of a conscious experience (e.g., what it feels like to “see red” vs. to “smell garlic”); the quantitative dimension corresponds to the intensity of a conscious experience; the hedonic dimension corresponds to the pleasantness, unpleasantness, or neutrality of a conscious experience; the temporal dimension corresponds to the duration of a conscious experience; and the spatial dimension corresponds to the egocentric perspective in which a conscious experience is embedded (I partly follow Cabanac, 2002—who, however, does not include the spatial dimension—in describing these dimensions of consciousness).

Each type of conscious experience results from a specific modulation and combination of the five dimensions: for instance, a perception has richer qualitative content than the meaning of a word; moods generally last longer and are of lower intensity than emotions.

The more complex a conscious experience is, the more cognitive components it requires, and the richer and more articulated the interplay of the five dimensions of consciousness becomes. Consider, for example, the difference between affects and emotions. In contrast to emotions, affects emerge very rapidly—sometimes within fractions of a second—and can fade just as swiftly. They often amount to no more than a basic sense that something is positive or negative, rely on minimal cognitive processing (sometimes no more than perceiving the stimulus and forming a single association), and may occur without physiological arousal (Baumeister et al., 2007). The reduced involvement of physiological arousal and cognitive processes in affects, as compared to emotions, which results in a weaker interplay among the five dimensions of consciousness, reflects in a less intense conscious experience.

Or consider the difference between emotions and motivations. Compared to motivations, emotions—while involving complex, multidimensional appraisal processes (Scherer, 2005, 2009) and specific modes of action readiness (Frijda, 1988)—are relatively primitive and largely stereotypical in their expressive and behavioral manifestations: they are more reactive and require less conscious thought. In contrast, motivations are complex structures that often involve deliberate cognitive evaluation and the formation of intentions (such as deciding on the steps to reach a goal); moreover, they gradually refine over the course of ontogeny through continuous and reciprocal interaction with the environment (Caprara, 1994). This greater involvement of cognitive components and processes in motivations, as compared to emotions, is reflected in conscious experiences characterized by a more elaborate integration of the five dimensions of consciousness.

While shaping every occurrence of conscious experience, the five dimensions of consciousness also contribute to building and shaping our sense of self. Every conscious experience is such precisely because it belongs to and refers to a self; it is fundamentally an experience of the self. Damasio (1998, p. 1880) makes this point very explicitly: “Consciousness occurs when we can generate, automatically, the sense that a given stimulus is being perceived in a personal perspective; the sense that the stimulus is owned by the organism involved in the perceiving; and, last but not least, the sense that the organism can act on the stimulus (or fail to do so), that is, the sense of agency.” In other words, consciousness and the subjective dimension are inextricably linked: they cannot exist without each other.

Our sense of self is characterized by some fundamental features, the main ones being the following (Marchetti, 2024):

(i) The sense of being an entity differentiated from other entities, which provides us with a sense of mineness or ownership;
(ii) The point of view or perspective from which any content is experienced;
(iii) The feeling of continuity—that is, the sense that our experience flows uninterruptedly; and
(iv) The feeling of unity, or a “single voice” (Damasio, 2010)—that is, the sense of being an organism composed of multiple parts interconnected in a unified way.

These features are not necessarily all present at the same time. Depending on the arousal state—for instance, conscious wakefulness, REM sleep, vegetative state, or near-death experience (Laureys, 2005; Laureys et al., 2009)—and the mode of self-consciousness—such as spatial, bodily, or cognitive self-consciousness (Millière et al., 2018; Millière, 2019)—some may be altered or even entirely absent.

Each of the five dimensions of conscious experience contributes, in its own way, to shaping the main features of the sense of self (Marchetti, 2022, 2024). The sense of being an entity differentiated from other entities is primarily made possible by the hedonic dimension; perspective, by the spatial dimension; the feeling of continuity, by the temporal dimension; and the feeling of unity, by the combined support of the qualitative, quantitative, temporal, and spatial dimensions.

In order to better understand the role that each of the five dimensions of consciousness plays in the formation of our knowledge of objects in the world and of our sense of self—how these dimensions condition and determine it, and the specific knowledge produced by each type of conscious experience, that is, how the knowledge generated by one type of consciousness differs from that generated by another—it is useful to undertake a deeper examination of the mechanisms underlying these five dimensions.

## 3 The attentional mechanisms underlying the dimensions of consciousness

According to the AME theory of consciousness, each of the five dimensions of consciousness (qualitative, quantitative, hedonic, temporal, spatial) can be explained by grounding them in the functioning of attention and its organ.

Attention is the most important—though not the sole—process responsible for the production of conscious experience (Marchetti, 2010, 2018, 2022). It is my hypothesis that there cannot be consciousness without attention (Marchetti, 2012). What we consciously experience is primarily determined by our attentional activity: where we focus it (internally or externally), how long we focus it, how we focus it (narrowly or widely), at what level of intensity, and so on.

I derive the hypothesis that there cannot be consciousness without attention from a number of theoretical and empirical observations. The idea that attention is strictly linked to consciousness is not new (James, 1890/1983; Posner, 1994). Empirical findings show a close correlation between attention and consciousness in visual perception (Liu et al., 2009), perception of time (Hicks et al., 1976, 1977; Brown, 1985; Shore et al., 2001; Coull et al., 2004), inattention blindness (Mack and Rock, 1998; Cartwright-Finch and Lavie, 2007), and change-blindness (Rensink et al., 1997). Although some scholars claim that consciousness can occur without attention (Lamme, 2003; Koch and Tsuchiya, 2006; van Boxtel et al., 2010; Bachmann, 2011), this claim can be rebutted on two main grounds. Firstly, as observed by several scholars (Srinivasan, 2008; Kouider et al., 2010; Marchetti, 2012; Pitts et al., 2018; Munévar, 2020; Noah and Mangun, 2020), this claim results from a misinterpretation of experimental data, which originates in a failure to consider the various forms and levels that attention (Nakayama and Mackeben, 1989; La Berge, 1995; Lavie, 1995; Pashler, 1998; Treisman, 2006; Demeyere and Humphreys, 2007; Koivisto et al., 2009; Alvarez, 2011; Chun et al., 2011; Tamber-Rosenau and Marois, 2016; Simione et al., 2019) and consciousness (Tulving, 1985; Edelman, 1989; Iwasaki, 1993; Bartolomeo, 2008; Vandekerckhove and Panksepp, 2009; Northoff, 2013; Northoff and Lamme, 2020) can assume. In fact, not all forms of attention produce the same kind of consciousness, and not all forms of consciousness are produced by the same kind of attention. For example, there can be kinds of conscious experience in the absence of top–down attention but in the presence of bottom-up attention; likewise, there can be kinds of conscious experience in the absence of a focal form of top–down attention but in the presence of a diffused form of top–down attention. Secondly, as observed by Cowan et al. (2024, 2025), claims that consciousness can occur without attention typically rest on a conceptual confusion between different components of attention. In particular, such claims tend to equate attention exclusively with the control of attention (e.g., top–down, executive, or report-related processes), while neglecting the role of the focus of attention. The control of attention, primarily mediated by frontal brain regions such as the dorsolateral prefrontal cortex (DLPFC) and the anterior cingulate cortex (ACC), can bring items into focus or prolong their duration. However, it is not the substrate of conscious experience itself, which is instead represented by the focus of attention centered in the intraparietal sulcus (IPS). The focus of attention acts as a capacity-limited hub, holding approximately 3–5 items, where information is integrated into a coherent representation that guides current thoughts and actions. Although conscious experience can occur in the absence of attentional control, it does not occur in the absence of an attentional focus. Any conscious content, on their account, must still fall within the focus of attention, understood as the limited-capacity state in which information is actively accessible and integrated. Accordingly, positions defending consciousness without attention are more accurately interpreted as defending consciousness without attentional control, rather than consciousness without attention per se.

Attention is deployed periodically (VanRullen et al., 2007; Bush and VanRullen, 2010; Landau and Fries, 2012; Fiebelkorn et al., 2013; Song et al., 2014; Zoefel and Sokoliuk, 2014; Dugué et al., 2015; VanRullen, 2016, 2018; Fiebelkorn and Kastner, 2019; Nakayama and Motoyoshi, 2019; Senoussi et al., 2019; Zalta et al., 2020).

A further hypothesis is that the attentional activity performed in each period modulates the energy level of the neural substrate—namely, the area of the organ of attention (henceforth, “aOA”)—that underpins this activity. *It is precisely this modulation that generates the phenomenal aspect of consciousness*. This hypothesis rests on the observation of the extreme effects that such modulation can produce, such as when the normal flow of attention is dramatically slowed down or even interrupted. Pain provides a paradigmatic example. A nociceptive signal seizes attentional resources, thereby inducing a modulation of the energy level of the aOA that, in cases of acute or chronic pain, may result in a disruption of the normal attentional flow (to the extent that, in order to restore the usual state, one must either redirect attention elsewhere or attempt to eliminate the source of pain) (Eccleston and Crombez, 1999; Legrain et al., 2009)—and this disruption is precisely what constitutes the experience of pain.

I speak of an “area” of the organ of attention because I explicitly refer to Tamber-Rosenau and Marois’ (2016) model of attention, which best aligns with the conception of an organ of attention. Tamber-Rosenau and Marois conceptualize attention as a structured mechanism composed of multiple levels and parts, each with distinct functional roles: a central level for abstract, cognitive processes; a mid-level containing priority maps that bias competition across representational formats and sensory modalities; and a peripheral level for sensory processes. According to this model, the organ of attention can be conceived as structured across various levels and parts, each underpinning one of these distinct roles.

Table 1 summarizes the principal hypotheses of the AME theory of consciousness presented above, along with the theoretical and empirical evidence supporting them. The table also highlights a major claim concerning emotional experience that is relevant to the present article and will be addressed in the section “An account of the emergence of emotion within the framework of the AME theory of consciousness.”

**Table 1:** Main hypotheses of the AME theory of consciousness and the relevant supporting theoretical and empirical evidence.

Main hypotheses of the AME theory of consciousness	Theoretical works and observations, and empirical findings supporting the hypotheses of the AME theory of consciousness
There cannot be consciousness without attention	The idea that attention is necessary for consciousness was advanced, among others, by James (1890/1983) and Posner (1994). Empirical findings show a close correlation between attention and consciousness in visual perception (Liu et al., 2009), perception of time (Hicks et al., 1976, 1977; Brown, 1985; Shore et al., 2001; Coull et al., 2004), inattention blindness (Mack and Rock, 1998; Cartwright-Finch and Lavie, 2007), and change-blindness (Rensink et al., 1997). According to several scholars (Srinivasan, 2008; Kouider et al., 2010; Marchetti, 2012; Pitts et al., 2018; Munévar, 2020; Noah and Mangun, 2020), claims that there can be consciousness without attention (Lamme, 2003; Koch and Tsuchiya, 2006; van Boxtel et al., 2010; Bachmann, 2011) result from a misinterpretation of experimental data, which originates from a failure to consider the various forms and levels that attention (Nakayama and Mackeben, 1989; La Berge, 1995; Lavie, 1995; Pashler, 1998; Treisman, 2006; Demeyere and Humphreys, 2007; Koivisto et al., 2009; Alvarez, 2011; Chun et al., 2011; Tamber-Rosenau and Marois, 2016; Simione et al., 2019) and consciousness (Tulving, 1985; Edelman, 1989; Iwasaki, 1993; Bartolomeo, 2008; Vandekerckhove and Panksepp, 2009; Northoff, 2013; Northoff and Lamme, 2020) can assume. Moreover, as Cowan et al. (2024, 2025) observe, claims that consciousness can occur without attention typically rely on an implicit identification of attention with its control mechanisms. Once a distinction is drawn between the control of attention and the focus of attention, these claims no longer entail consciousness without attention in the relevant sense, but rather consciousness without attentional control.
Attentional activity is made possible by the neural energy provided by the organ of attention (OA)	Kahneman’s (1973) theory puts forward the idea that attention is based on an energy pool. Various experimental paradigms (dual-task, attentional blink, psychological refractory period, visual search) show that attentional capacity is limited, that there is a limit to increasing mental processing capacity by increasing mental effort and arousal (Styles, 1997; Pashler, 1998), and that the possibility of sharing attention is constrained by task demands: when one task requires more resources, less capacity remains available for other tasks (Lavie, 1995). These findings indicate that attentional resources are based on a limited energy pool.
Attentional activity modulates the energy level of the aOA. This modulation generates the phenomenal aspect of consciousness	To the best of my knowledge, there are neither theoretical works nor empirical observations explicitly supporting this hypothesis. However, the works of de Biran (1802, specifically the *Introduction*) and Valéry (1973) contain conceptually relevant suggestions. Notably, Paul Valéry observes that sensation is a variation in the state of energy of a closed system: “Sensation does not consist so much in an introduction of something from the outside, as in an intervention—that is, an inner transformation (of energy) made possible by an external modification, a variation in the state of a closed system (…) sensation is due to some kind of disequilibrium (…) sensation is what occurs between two states of equilibrium” (translated from the Italian version, 1988, pp. 411–412).
The phenomenal aspect of consciousness supplies the agent with a sense of self	Several theories support the view that conscious experience contributes to the generation of the sense of self, or of some of its main aspects: for example, Damasio (1999, 2010), Legrand (2006, 2007), Legrand and Ruby (2009), Williford et al. (2012, 2018), and Berkovich-Ohana and Glicksohn (2014). However, this view is not yet directly supported by empirical evidence. Empirical findings mostly support this view indirectly, by showing how the sense of self can be modulated or altered by a wide range of means that affect conscious states, including meditation (Berkovich-Ohana et al., 2013; Jerath et al., 2016; Fingelkurts et al., 2016a, 2016b, 2020; Millière et al., 2018), hypnosis (Kallio and Revonsuo, 2003), perceptual deprivation (Glicksohn and Ben-Soussan, 2020), pharmacological means (Millière et al., 2018; Deane, 2020), induced illusory own-body perceptions (Ionta et al., 2011; Petkova et al., 2011; Blanke, 2012; Pfeiffer et al., 2013). For a review, see Marchetti (2024).
Emotional experience is essentially based on an act of focusing one’s attention on one’s own internal state	Negative affect increases self-focus (Salovey, 1992; Sedikides, 1992; Wood et al., 1990); positive affect also boosts self-focus, but only if the situation is not demanding (if the situation is demanding, positive affect leads to a decrease in self-focus) (Abele et al., 2005)

According to the AME theory of consciousness, each dimension of the phenomenal aspect of consciousness can be explained by a specific aspect of the modulation of the energy level of the aOA:

(i) The qualitative dimension is defined by the specific aOA that underpins, and is consequently modulated, by attentional activity;
(ii) The quantitative dimension is defined by the amplitude of the modulation;
(iii) The hedonic dimension is defined by the direction of the modulation—namely, whether the energy level of the aOA moves toward or away from the set-point at which it is set—and by the speed at which the modulation occurs. Set-points establish the optimal reference energy level for the functioning of the aOA that underpins the attentional activity being performed. Experiences of pleasure and displeasure arise when the energy level moves toward or away from the set-point, respectively, and their intensity increases with the speed of this movement. Neutral experiences—a state characterized by physiological normality and indifference toward the environment—occur when the energy level fluctuates within a tolerable range of the set-point. Set-points can—up to a certain limit—be adjusted by the agent according to plans, goals, motivations, etc.

I derive the idea of a set-point as an optimal reference energy level for the functioning of the aOA from studies on the functioning of homeostatic systems (Cabanac, 1971, 1979, 2006; Cabanac and Russek, 2000). As Cabanac (2006, pp. 1338–1341) explains:

“A set point is an information input that may be determined by an external signal to which the regulated variable is compared or may be determined by the structural characteristics of the system itself (…) Set point is the value defended by a regulation. In the absence of external perturbation the regulated variable stabilizes at the set point of the system. (…) For example, temperature regulation defends a core temperature close to 37 °C; glucose regulation defends a blood glucose concentration close to 1 g/L, and so forth. The difference between the actual value of a regulated variable and its set point is the *error signal*. The error signal triggers correcting defense responses opposing it. For example, when core body temperature is below set point, temperature regulation opposes such hypothermia with behavior and shivering (the latter only in endothermic animals) (…) Set points may be constant as is the case with calcemia regulation, but they are most often adjustable from inherent internal signals, cyclically, or unidirectionally during aging, and under the influence of peripheral sensory signals.”

In this view, a set-point is a *neutral* baseline that defines what counts as deviation and does not represent a *positive* state in itself. Rather, it serves to define what counts as restoration (pleasant) or perturbation (unpleasant). Therefore, the hedonic dimension does not depend on the absolute value of the set-point, but on the direction of the deviation relative to it: (a) movements toward the set-point are experienced as positive/pleasant because they signal a return to equilibrium (“a stimulus is pleasant when it facilitates the return of internal temperature to its normal value,” Cabanac, 1971, p. 1105^1^); (b) movements away from the set- point are experienced as negative/unpleasant because they signal a disruption of equilibrium; (c) fluctuations within a small tolerance range around the set-point correspond to neutral experience, because they do not meaningfully alter the organism’s internal equilibrium.

(i) The temporal dimension is determined by the periodic nature of the attentional activity—a nature that limits the duration of the modulation and, consequently, of any conscious experience. This limit is overcome primarily with the support of working memory;
(ii) The spatial dimension is grounded in the egocentric nature of attention: every attentional pulse originates from a single point within the body and is directed toward an object, which, according to Merker (2013, p. 9), “is located at the proximal most end of any line of sight or equivalent line of attentional focus.” The trajectory followed by attention is mirrored in the spatial boundaries of the aOA: it starts from the point where attention originates and continues to the point where its deployment stops. The egocentric perspective constitutes the foundational framework upon which the allocentric perspective can be constructed, relying on additional capacities such as translocating the egocentric perspective to external objects, creating spatial maps, and remembering spatial information, which require the support of additional cognitive mechanisms such as working memory and procedural memory. The spatial dimension is also defined by the scope of attention, that is, by the degree to which information is focused narrowly or broadly.

The primary determinant of the modulation of the energy level of the aOA is the object of attention—that is, what attention focuses on: an external object, one’s body, a memory, an idea, and so on. Once focused, each object modulates the energy level of the aOA in a specific way (e.g., a stark light produces a larger modulation amplitude than a faint light). It should be noted that, in theory, attention can be deployed even in the absence of an object, as if in a “pure” or “suspended” state—such as when one is on the lookout for or expecting something that has not yet appeared (see Ceccato and Zonta, 1980, p. 51: “Pure attention is the one with which we respond to words like ‘Look out!’ or ‘Look!’”). Usually, however, attention is directed toward some object. In this sense, we can say that conscious experience, most of the time, provides information about its object and how the object relates to us.

Importantly, the object of attention is always provided by and through our bodily–neural system, what I call “the Self” (Marchetti, 2022)—that is, our body and brain, excluding attention and its organ (I capitalize “Self” to avoid confusion with the sense of self: whereas the Self is a physical entity, the sense of self is a conscious experience) (on the distinction between the sense of self and its underlying brain basis, see also Davey and Harrison, 2022).

The Self is primarily expressed via the central and peripheral nervous systems, which map our body, environment, and interactions with the environment (Marchetti, 2018), and embodies all the competencies and abilities—physical, social, linguistic, and so on—that we innately possess and acquire throughout life. It operates according to a fundamental principle or goal, which underlies all other principles: to stay alive. Operationally, this principle can be formulated as follows: “operate in order to continue to operate” (Marchetti, 2010). It represents the vital instinct, the algorithm of life, present even in the simplest cells (Damasio, 2010). This principle is primarily instantiated in a hierarchy of values, among which biological values (e.g., homeostasis) occupy a central and foundational role. Upon these values, other kinds of values (e.g., cultural) may be developed throughout the agent’s life. These values determine what is relevant and meaningful for the agent and guide the development of the Self.

The functioning of the Self is primarily non-conscious and unconscious. However, one of its components—working memory—typically renders the processes or elements of the Self preconscious, that is, immediately accessible for conscious processing (Dehaene et al., 2006). In this light, the activity of working memory is particularly important because, by enabling the flexible assembly of multiple attentional pulses, it allows us to consciously experience more than what a single attentional pulse would afford.

It is the Self that supplies the contents of conscious experience, whether perceptible ones, such as “yellow” and “cat,” or intangible ones, such as memories, ideas, and emotions. Even when the object is external to the body, the sensory information concerning it is provided by the Self. In the case of vision, for example, sensory signals are generated by the photoreceptors of the retina, which transduce light into neural activity. This activity is then progressively transmitted and transformed through successive stages—via the optic nerve, the optic chiasm, and thalamic relays—before reaching primary and associative cortical areas. Therefore, attention to the object is necessarily mediated by the body and the nervous system.

A second important factor in modulating the energy level of the aOA is the amount of energy supplied to it by the organism. Like any other organ, the functioning of the organ of attention depends on the energy it receives from the organism. To operate properly, the organ of attention requires a certain amount of energy. The energy supplied by the organism to the organ of attention fluctuates with conditions such as physical and mental activity, energy reserves, overall health, and the intake of stimulants. These fluctuations affect the individual’s overall arousal state (or wakefulness: Laureys, 2005; Laureys et al., 2009), which in turn shapes his conscious experiences. For example, intensive physical activity may cause a drastic reduction in the organism’s overall energy, thereby making less energy available to the organ of attention. As a result, the energy of the organ of attention may take longer—or even fail—to be restored to its optimal level. This may give rise to feelings such as tiredness, fatigue, discomfort, and even drowsiness.

Another important factor that must be taken into consideration when addressing the modulation of the energy level of the aOA is, as mentioned above, the possibility that the individual has to flexibly adjust—within certain limits—the set-point of the aOA according to his needs, plans, goals, motivations, attitudes, and expectations. Different needs, goals, motivations, attitudes, and expectations may entail distinct set-points, in addition to engaging different areas of the organ of attention. For example, an individual who is forced to perform a job that is ungratifying or that he dislikes will, in an attempt to adapt, modify the set-point to reduce the impact of discomfort, accepting a certain degree of annoyance as the baseline.

**Figure 1:**
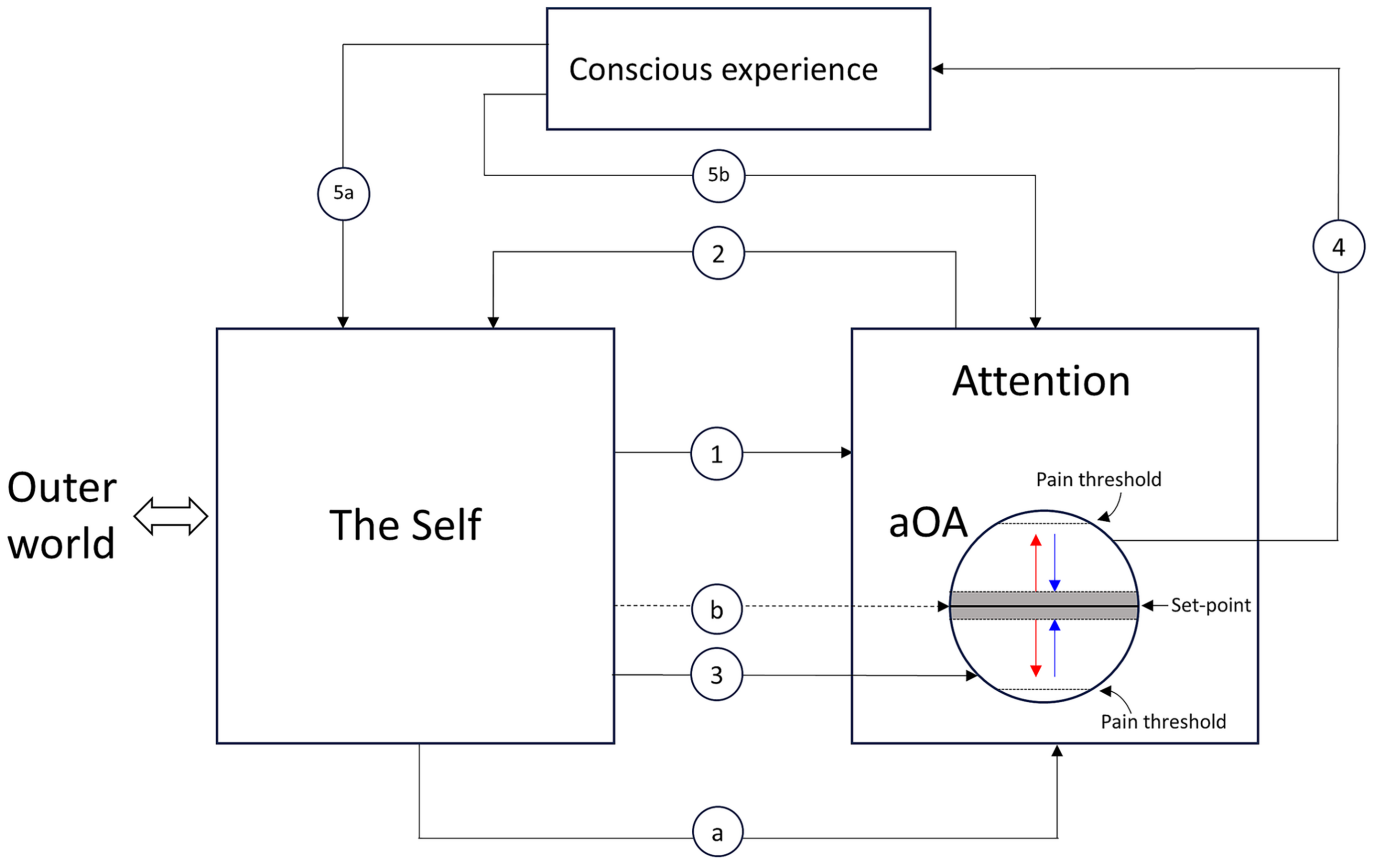
The main components, their functional relations, and the steps involved in the modulation of the area of the organ of attention (aOA). The Self, which comprises the body and the brain but not attention, interacts with the outer world and provides goal-directed and stimulus-driven instructions to attention (arrow line 1). Upon receiving instructions from the Self, attention focuses on its object (arrow line 2), supported by the specific aOA that underpins it. Attentional activity entails a modulation of the energy level of the aOA (arrow line 3). The modulation of the energy level of the aOA produces conscious experience (arrow line 4). Fluctuations of the energy level within the threshold area (shown as a gray zone) surrounding the set-point generate a neutral experience (e.g., indifference, neutrality, normality). Outside the threshold area surrounding the set-point, when the energy level moves away from the set-point (red arrows), unpleasant experiences occur; when the energy level moves toward the set-point (blue arrows), pleasant experiences occur. Conscious experience modifies the Self, triggers unconscious processes, induces modifications of the cultural values regulating the Self (arrow line 5a), guides attention (arrow line 5b), and, through it, triggers intentional actions. Arrow line “a” represents the energy flow from the organism to the organ of attention. Arrow line “b” represents the possibility of flexibly adjusting the aOA’s set-point according to an individual’s needs, plans, goals, motivations, attitudes, and expectations. The entire process—beginning with arrow line 1 and concluding with arrow lines 5a and 5b—is repeated continuously, cycling anew each time it reaches completion.

Figure 1 shows the main components, their functional relations, and the steps involved in the modulation of the aOA.

The main components are the Self, the outer world, attention, and consciousness. The Self provides goal-directed or stimulus-driven instructions to attention (arrow line 1): what, how, where, and for how long to focus (I chose the instructions from the Self as a starting point for the sake of simplicity, though, as will be shown, they may also come directly from consciousness). These instructions are the primary determinants of the qualitative, spatial, and temporal dimensions of conscious experience. For example, if we pay attention to the taste of what we are eating, we will have taste perceptions rather than visual ones, and these perceptions will be spatially located inside our mouth rather than outside our body.

The Self continuously interacts with the outer world, which provides it with the majority of inputs and serves as the primary means to satisfy its (physical, social, economic, etc.) needs, while also representing the main challenges to its survival.

Attention is composed of multiple levels and parts, each with distinct functional roles (Tamber-Rosenau and Marois, 2016). Upon receiving instructions, attention focuses on its object (arrow line 2), supported by the specific aOA that underpins it. As explained earlier, the object of attention is always provided by the Self, in the sense that even external objects must first be detected by the body’s sense organs and transduced into neural signals.

According to my hypothesis (Marchetti, 2018, 2022, 2024), performing attentional activity entails a modulation of the energy level within its neural substrate, the aOA (arrow line 3). The modulation is characterized by four main dimensions: amplitude, polarity, direction, and speed (represented by the arrow lines inside the aOA). Regarding amplitude, it can range from a negligible minimum to a maximum. The negligible minimum is defined by the threshold area shown in Figure 1 as a gray zone surrounding the set-point, within which fluctuations in the energy level generate a neutral experience—a feeling of “indifference,” “normality,” or “neutrality” toward the object. The maximum is defined by the two pain thresholds illustrated in Figure 1: experiences become painful when the energy level moves beyond these thresholds. Regarding polarity, it can be positive or negative. Regarding direction, it can move either toward or away from the set-point: in the former case, pleasant experiences occur, while in the latter, unpleasant ones occur. Finally, regarding speed, it can move quickly, slowly, or at an intermediate pace, reaching the final value sooner or later (Carver and Scheier, 1990).

The modulation of the energy level of the aOA produces conscious experience (arrow line 4). In Figure 1, conscious experience is represented as a component—alongside the Self and attention—rather than as a mere outcome or a passive conduit. As we saw earlier, the formation of conscious experience entails the simultaneous emergence of the sense of self, which establishes a distinct and hierarchically higher level of processing than that of the Self and attention. This implies that consciousness not only modifies the Self by triggering unconscious processes and inducing changes in the cultural values regulating the Self (arrow line 5a), but can also autonomously and directly guide attention and, through it, trigger intentional actions (arrow line 5b).

These modifications of the Self thus produced provide the necessary input to start a new attention cycle (arrow line 1). It is important to note that, in each new attentional cycle, modulation proceeds from the level attained in the previous period. This allows the subject, among other things, to verify whether, and to what extent, the activities previously undertaken to restore the optimal energy level in the aOA—or to maintain the alteration of that level—have produced the desired effect. I say “to maintain the alteration of that level” because, while we usually tend to minimize and extinguish negative feelings (by restoring the energy level of the aOA to its set-point), it may also happen that we try to prolong positive feelings (which, however, as noted by Carver and Scheier, 1990, cannot be sustained for long).

Figure 1 also shows two additional arrow lines—“a” and “b”: arrow line “a” represents the energy flow from the organism to the organ of attention; arrow line “b” represents the possibility of flexibly adjusting the aOA’s set-point according to an individual’s needs, plans, goals, motivations, attitudes, and expectations.

To exemplify how amplitude, polarity, direction, and speed of modulation collectively shape phenomenal experience, consider thermal sensation. The set-point for ambient temperature considered comfortable by most people generally lies around 20 °C. When the perceived temperature is close to this comfort set-point, the modulation of energy within the aOA remains inside the threshold range of the set-point, resulting in a neutral experience—a feeling of normality. However, entering an environment that unexpectedly turns out to be warmer than the set-point, for example above 30 °C, or colder than the set-point, for example 10 °C, generates a sudden deviation of the energy level well beyond the threshold range of the set-point—above or below the set-point, respectively—resulting in an unpleasant sensation.

Similar considerations can be made for any other type of stimulus or object. Each of us has his own idea of “freedom,” which means that each of us defines this idea with a personal and specific set-point, and that my set-point differs from yours. It can therefore happen that, when I observe how your idea of freedom is expressed in your behavior and speech, I judge it either insufficiently liberal (or even conservative) or, conversely, too liberal (if not anarchic) compared to mine. This judgment is made possible precisely by the extent and polarity of the deviation that the energy level of my aOA undergoes—below or above the set-point, respectively—because of your behavior and speech.

As the example of “freedom” illustrates, the attentional mechanism that I propose as the main determinant of the production of conscious experience makes it possible to account for an essential property of consciousness: namely, that objects and stimuli of various kinds—physical objects, living beings, events, abstract ideas, or other entities—within consciousness all acquire an appearance that renders them commensurable with one another. The processing carried out by the attentional mechanism translates objects of differing natures into the “words” of a single “common language”: the language of consciousness. In consciousness, the various objects, while remaining clearly distinct from one another (a concrete object is something quite different from an abstract idea), can nonetheless all be treated in the same way: they can be associated, combined, compared, placed into various relations, and so forth (an abstract idea can be clarified by means of a concrete object—such as a balance representing the idea of justice; conversely, a concrete object may evoke an abstract idea, and so on). It is this common language that makes it possible to subsume the various dimensions of life—physical, biological, social, economic, political, artistic, and so forth—under a common conscious experiential dimension, thus making them comparable and differentiable.

## 4 The components of emotion

Emotions are conscious states characterized by a high degree of informational complexity. An appreciation of the complexity of the information conveyed by emotions can be gained by enumerating the principal kinds of information they transmit, as identified by scholars in the field of emotion studies (Scherer, 2005, 2009; Baumeister et al., 2007; Grandjean et al., 2008; Izard, 2010; Sander, 2013; Scherer and Moors, 2019). Emotions:

(i) Inform us that our self has entered a state of disequilibrium;
(ii) Indicate the valence of the disequilibrium (pleasant or unpleasant) and its intensity (strong or weak);
(iii) Signal that the disequilibrium has been caused by some object—whether external (events, people, objects) or internal (thoughts, memories)—and typically identify which object it is (though the object may remain unknown, resulting in the feeling of a vague emotion);
(iv) Signal the nature of the disequilibrium—whether it is due to the novelty of the object, its unexpectedness, or some other cause;
(v) Indicate the relationship between the disequilibrium and potentially relevant social norms and values;
(vi) Inform us about our coping potential—how well we can cope with the disequilibrium;
(vii) Guide us—by directing our attention and eliciting specific physiological responses and action tendencies and readiness—toward actions aimed at restoring the original equilibrium, creating a new equilibrium that is acceptable to us, or maintaining the disequilibrium;
(viii) Help us communicate our emotional state to others, facilitating social interaction;
(xi) Inform us about the adequacy of our behavior, prompt us to reevaluate our decision processes, and help us extract lessons on how a different course of action might have yielded better outcomes—essentially functioning as an instructive feedback system.

Most theories of emotion—including appraisal theories (Scherer, 1984, 2005; Smith and Ellsworth, 1985; Sander et al., 2005, 2018; Scherer and Moors, 2019; Moors, 2020), constructivist/constructionist theories (Schachter and Singer, 1962; Russell, 2003; Barrett, 2006; Lindquist et al., 2015), and adaptational and neurophysiological theories (Panksepp, 1998, 2005; Damasio, 1999, 2010; Lang and Bradley, 2010)—appear to converge (Sander, 2013) on the view that the complex informational content of emotions arises from the interaction of various components. These components can ideally be grouped into three main groups: core affect, appraisal processes, and physiological and behavioral manifestations (scholars often use the expression “emotional responses,” but I prefer “physiological and behavioral manifestations” since some of these manifestations can precede the actual emotional state). Each of these components takes shape in different ways across the five dimensions of consciousness.

### 4.1 Core affect

As Russell (2003, p. 149) explains, “core affect is a continuous assessment of one’s current state, and it affects other psychological processes accordingly.” Core affect is taken to reflect the valence of an experience—that is, the extent to which it is pleasant or unpleasant (Russell and Barrett, 1999; Russell, 2003; Barrett et al., 2007; Barrett and Bliss-Moreau, 2009)—and the associated level of arousal or activation—that is, the experience of feeling energetic versus enervated (even though, some psychologists question whether arousal constitutes a truly fundamental aspect of core affective states; see Barrett et al., 2007).

Both dimensions of core affect—valence and arousal—are represented in the AME theory of consciousness. Valence is determined by the direction, polarity and speed of the modulation of the energy level of the aOA. Arousal is determined both by the amount of energy the organism supplies to the organ of attention and by the amplitude of the modulation induced by the object.

As we saw earlier, all forms of conscious experience are characterized by the two dimensions of core affect, making it *ubiquitous* (Russell, 2003; Barrett et al., 2007). A person always has core affect and is always in some state of core affect, even if that state is neutral (Cabanac, 1996, 2002; Russell and Barrett, 1999, p. 806; Russell, 2003, p. 148). Consequently, every mental state is intrinsically infused with affective content (Barrett and Bliss-Moreau, 2009, p. 178), and all objects and events—whether real, imagined, remembered, or anticipated—enter consciousness already affectively interpreted (Russell, 2003).

Core affect is also *primitive* in nature. According to Russell (2003, p. 148), core affect “can exist without being labeled, interpreted, or attributed to any cause. As an analogy, consider felt body temperature. You can note it whenever you want. Extremes can become very salient. Felt temperature exists prior to such words as hot or cold, prior to the concept of temperature, either in folk or scientific theory, and prior to any attribution about what is making you hot or cold.”

Core affect is also *universal*. As Barrett et al. (2007, pp. 377–378) observe, (i) the capacity to experience pleasure and displeasure is universal to all humans, (ii) experiences of pleasure and displeasure are present at birth, and (iii) all known human languages have words to communicate pleasure and displeasure (Wierzbicka, 1992; Russell, 2003).

Core affect contributes to how a person experiences and responds to the environment primarily by influencing how he reacts to a certain stimulus or event. A person who feels bad in a certain context or undergoes an unpleasant situation will likely try to avoid it. Conversely, a person who feels good will seek to prolong or repeat the experience. In this perspective, core affect represents “a form of affective responding that functions as a kind of core knowledge about whether objects or events are helpful or harmful, rewarding or threatening, calling for acceptance or rejection” (Barrett et al., 2007, p. 377).

Core affect also influences memory and learning. As demonstrated by Pavlovian or classical conditioning, neutral stimuli that are repeatedly paired with a stimulus (the unconditioned stimulus) that has the capacity to perturb core affect, such as a loud noise or an electric shock, acquire the capacity to elicit the same affective response as the unconditioned stimulus (Barrett and Bliss-Moreau, 2009). Moreover, as Bower (1992, p. 14) observes: “a strong affective reaction after an event also causes the reactivation, rehearsal, or ‘mulling over’ in working memory of the encoded version of that event (…) Such rehearsal enhances the degree of learning of whatever has been encoded of the emotional experience.”

Despite its centrality to emotional experience, core affect is not sufficient to characterize it. On the one hand, core affect is common to affective experiences other than emotions, such as moods and sentiments, as well as to events that are not considered prototypical emotional episodes—such as feeling miserable from a low-grade infection, feeling tension at the end of a stressful day, or feeling serenity on a lazy summer day spent at the shore (Russell and Barrett, 1999, p. 806). On the other hand, the same core affect (e.g., feeling displeased) can lead to different emotions, such as anger, sadness, or frustration, depending on how the person interprets the situation. Therefore, to elicit an emotion, core affect must integrate with other components. Most scholars agree that, because emotions are directed at an object, these components should enable the individual to cognitively and conceptually appraise the object in relation to his personal values, goals, desires, and expectations, as well as contextual factors, social and cultural norms.

### 4.2 Appraisal processes

According to Scherer (2009), the cognitive appraisal of an object is based on several basic criteria, which allow the individual to adaptively react to it. The criteria can be classified into four major dimensions: (i) Relevance: how relevant is the event to the individual? Does it directly affect the individual or their social reference group? (ii) Implication: what are the implications or consequences of this event and how do they affect the individual’s wellbeing and immediate or long-term goals? (iii) Coping potential: how well can the individual cope with or adjust to these consequences? (iv) Normative significance: what is the significance of this event for the individual’s self-concept and in relation to social norms and values? These criteria include aspects such as the novelty or familiarity of the event or stimulus, its intrinsic pleasantness or unpleasantness; its significance for the individual’s needs or goals; its perceived causes, its conduciveness to satisfying a need or achieving a goal.

At first glance, the set of appraisal criteria proposed by Scherer may seem somewhat extensive, complex, or even unnecessary. However, to appreciate its adaptive usefulness, consider the emotions elicited by the sight of a bar of chocolate. While the chocolate may be intrinsically pleasant to a person, it can also elicit negative feelings if that person is on a diet or has already eaten too much. Conversely, a medicine can be appraised as beneficial for one’s health and can therefore elicit a positive feeling, even though it smells and tastes unpleasant.

The results of the evaluations based on the set of appraisal criteria—evaluations that can occur unconsciously, as they often rely on evolutionary mechanisms or past experiences—are reflected in various ways across the main dimensions of consciousness. For example, an event that is highly relevant to the individual, or one that the individual can hardly cope with, will require more attention and, consequently, will be experienced more intensively, than an event that is less relevant or easier to cope with. Likewise, an event that is judged to be persistent will involve sustained attention and working memory to a greater extent than an event judged to be brief—likely prolonging the emotional experience.

Just as core affect alone cannot fully account for why people feel the way they do when experiencing an emotion, neither can cognitive appraisal processes alone (Barrett et al., 2007, p. 380). This is demonstrated not only by the variability in emotional intensity and qualia when people experience the same situation, and by the impact of cultural and social factors, but also by the role of physiological and behavioral manifestations in shaping emotions.

### 4.3 Physiological and behavioral manifestations

Typical physiological and behavioral manifestations include physiological reactions, behavioral expressions, and states of action tendencies and readiness.

Examples of physiological reactions triggered by emotional stimuli include changes in heart rate, blood pressure, hormone levels, skin conductance, and pupil dilation (these physiological responses, along with behavioral, neuronal, and self-reported data, are used in the context of emotion studies to determine an individual’s arousal or activation level induced by emotional stimuli).

Typical behavioral expressions associated with emotions are facial expressions, such as smiling when happy or frowning with anger (Ekman, 2003; George, 2013).

Emotions also usually result in specific states of action tendencies and readiness, such as approaching, avoiding, dominating, and submitting—that is, states of action that prepare and orient the body to respond to the possibilities and challenges that matter most to the organism at the time (Frijda, 1988, 2009a, 2009b). According to Frijda (1988), states of action tendencies and readiness help unambiguously define and distinguish several emotions, particularly those considered primary.

## 5 An account of the emergence of emotion within the framework of the AME theory of consciousness

In the previous section, we saw that emotions provide us with specific and complex informative content that tells us how objects relate to us, whether and in what way they matter to us in the present, and how we can cope with them. This content arises from the interaction of three main components—core affect, cognitive appraisal processes, and physiological and behavioral manifestations.

Let us now examine how the AME theory of consciousness can explain the generation of the complex informational content of emotions through the interaction of the three components.

Among the various models proposed to account for how emotions develop, I will refer to Baumeister et al.’s (2007) model, which, in addition to encompassing the various cases of emotional experience, lends itself well to a description in terms of the succession and interaction of conscious and unconscious states and is therefore the most suitable for applying the AME theory of consciousness.

Baumeister et al. (2007) describe the emergence and development of emotions as follows. Initially, the subject has a conscious experience of a given object (physical object, event, behavior, etc.), which—among other pieces of information—includes an affective component (e.g., liking, disliking, or indifference). As Baumeister et al. (2007, p. 169) observe, affect often consists “of no more than a simple feeling that something is good or bad, to be approached or avoided. It does not rest on elaborate cognitive processing: the feeling of liking or disliking some stimulus may require nothing more than perceiving the stimulus and making one association.” This simple feeling informs the Self (via the information flow indicated by arrow 5a in Figure 1), which, if certain conditions are met, generates intermediate conscious experiences—such as cognitive appraisal and increased arousal—that lead the subject to consciously experience the emotion proper. The conscious experience of the emotion replaces that of the object that initially elicited it and engages all of the subject’s attention and energy, permeating his existence entirely. The conscious emotional experience, in turn, fosters further cognitive processing (via the information flow indicated by arrow 5a in Figure 1) in an attempt to enable the subject to resolve the imbalance signaled by the emotion. This conscious emotional experience may prevail for an extended period over the conscious experience of the object that elicited the emotion, as well as over other objects, at times alternating with them, as observed in cases of bereavement.

Some clarifications are in order concerning this model of the emergence of an emotion before we proceed to analyze the fundamental mechanisms that make the experience of the emotion proper possible.

First, as I specified, the Self promotes the emergence of emotion—upon receiving the affective information elicited by the object—provided that “certain conditions are met.” Indeed, even though the affective dimension characterizes and constitutes all forms of conscious experience, it does not always give rise to an emotional experience. In my view, for an emotional experience to arise, the affective information must signal either a high amplitude in the modulation of the energy level of the aOA, a high speed in that modulation, or a difficulty in restoring the level to its set-point value^2^. In this regard, it is necessary to highlight that, for an object to be able to elicit an emotional experience, it must in turn have an emotional meaning for the person. No object is, in itself, capable of eliciting an emotion: much depends on the person’s interests, needs, expectations, goals, motivations, mood, personality traits, culture, and so on. As Frijda’s (1988, p. 349) *Law of Situational Meaning* states, “It is meanings and the subject’s appraisals that count—that is, the relationship between events and the subject’s concerns, and not events as such.” Indeed, the same object can elicit any kind of emotion—or no emotion at all; an emotion can be elicited by any object; and no object must always and necessarily elicit a specific emotion.^3^ This dependence of an object’s emotional meaning on subjective factors such as interests, needs, and motivations is accounted for in the AME theory of consciousness by the flexible way in which the set-point of the aOA can be adjusted according to the person’s needs, plans, etc. (see arrow line “b” in Figure 1), and by the variations in the amount of energy supplied to the organ of attention by the organism (see arrow line “a” in Figure 1).

**Figure 2:**
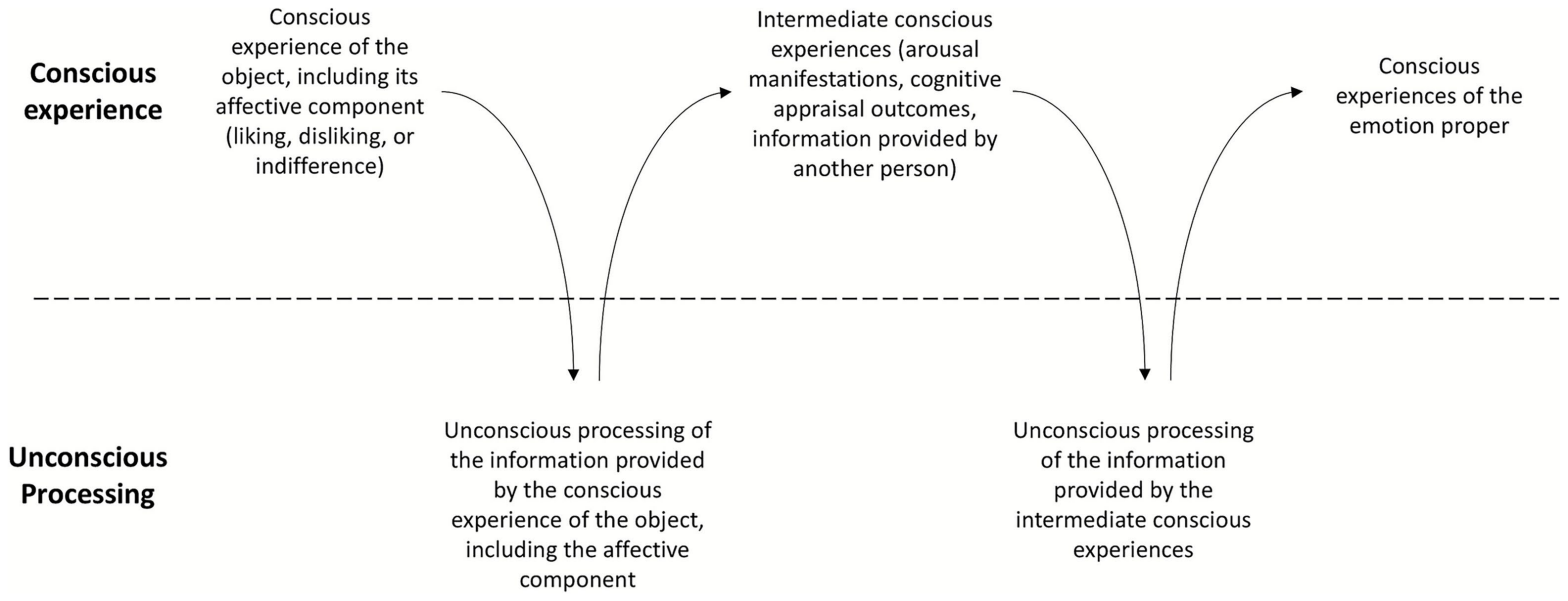
The alternating sequence of conscious experiences and unconscious processes through which the emotion proper emerges.

Second, the transition from one kind of experience to another—from the experience of the object to that of the emotion, passing through intermediate experiences in which the subject undergoes an increase in arousal (changes in heart rate, blood pressure, sweating, and so forth) and/or appraises the object’s relevance and implications for himself, his ability to cope with it, and so forth—is primarily, albeit not exclusively, determined and guided by the unconscious processes taking place within the Self (I say “primarily, albeit not exclusively” because, as the information flow indicated by arrow 5b in Figure 1 shows, conscious experience too can guide attention and, through it, trigger new conscious experiences). These unconscious processes are, in turn, mainly triggered by information originating from consciousness (the information flow indicated by arrow 5a in Figure 1). In other words, conscious experiences are to a large extent determined by the unconscious processes occurring within the Self. This alternating sequence of conscious and unconscious processes that leads to the emergence of the emotion proper can be represented as shown in Figure 2. Figure 2 is a simplified example of what may actually take place. It may happen, for instance, that before promoting an emotional experience, the Self requires the conscious experience of the object to occur repeatedly. Likewise, several intermediate conscious and unconscious processes may be necessary before the emotion emerges.

Third, this model operates independently of whether an emotion is triggered by cognitive appraisal (as claimed, for example, by Lazarus, 1982), by affect/increased arousal (as claimed by Zajonc, 1984), or by someone else directing the subject’s attention to his bodily changes (e.g., blushing on the cheeks and sweating), thereby causing him to experience the emotion: the transition from the conscious experience of the object to that of the emotion can be driven by any of these intermediate conscious and unconscious processes (see Figure 2).

Coming now to the fundamental mechanisms that make the experience of the emotion proper possible, we may note that the arising and predominance of the conscious experience of emotion—over that of the object—are made possible by, and correspond to, a drastic change in the object of attention: namely, from the object that initially elicited the emotion to the sense of self as an object. When a person experiences an emotion, he is first and foremost conscious of his own state and of how that state has changed from what it was before—that is, he experiences the emotion as the perception of this inner change. As Reeve (2022, pp. 257–258) observes, emotions “arise in the simplest sense when we bring our attention to our internal states, because in doing so we are taking a perspective on ourselves.”

The predominance of the conscious experience of emotion is evident in the total hold it takes over a person’s mind and body: once the person starts experiencing the emotion, he cannot resist it and becomes, as it were, its slave. Think, for example, of how emotions such as jealousy, anger, envy, terror, but also surprise and joy, invade our consciousness with their presence, almost preventing us from thinking of anything else except of them, pushing us to do everything we can to free ourselves from them when they are negative, or to try to hold on to them as long as possible when they are positive. In sum, all emotions are “self-relevant,” as Tangney observes (2005, p. 384).

The shift in the object of attention—from the object that initially elicited the emotion to the sense of self—is facilitated by the intermediate conscious and unconscious processes triggered by the affective component of the conscious experience of the object (see Figure 2). As noted, these intermediate processes can take various forms: increased arousal, cognitive appraisal, information provided by another person that directs the subject’s attention to his bodily changes, or a combination of these. In all these cases, the person’s attention is drawn to himself. Increased arousal leads the person to notice, for example, that he is sweating, that his voice has changed, or that his heart rate has accelerated—all physiological and behavioral manifestations that cause him to focus on his own body, movements, gestures, and so on, inevitably bringing the sense of self to the foreground of his consciousness (for experimental confirmation, see for example Wegner and Giuliano, 1980). Likewise, cognitive appraisal leads the person to evaluate the relevance of the object to himself—what consequences it might have for him, how it affects his wellbeing or his goals, whether he can cope with it, and so forth—all processes that help the subject focus on his self. As Tangney (2005, p. 384) observes:

“In the language of appraisal theory (…), we experience emotions when we judge that events have positive or negative significance for our wellbeing. The specific type of emotional response is shaped by both primary appraisal of the positive versus negative implications of the event for the individual and by secondary appraisals (e.g., of one’s ability to cope with the events). But all emotions arise from events that in some way have relevance for oneself.”

The same holds when a person’s attention is brought to his or her own bodily changes by someone else.

In terms of the AME theory of consciousness, the change of the object of attention from the object that initially elicited the emotion to the sense of self is reflected in the activation of a different aOA and, consequently, in the adoption of a different set-point. What the new set-point indicates is something related to the sense of self: it may be the sense of self understood in its entirety, or a specific dimension of it—cognitive, affective, social, or moral—that is, at that moment, significant or predominant; however, it is no longer something that concerns the object itself, such as its shape, size, color, position in space, and so on.

Depending on the context, the subject may direct his attention to a certain aspect or dimension of his sense of self rather than to another, and consequently activate a specific aOA. This results in the adoption of the set-point that reflects the focused aspect or dimension of the sense of self. For instance, if the context leads the person to focus his attention on his body or a part of it—for example, when he faces an object that puts his physical safety at risk—then a set-point will be adopted that serves to determine and signal the impact that the object has on the person’s state of physical wellbeing and bodily integrity. Likewise, if the context leads the person to focus his attention on his affective and sentimental sphere—because his partner has left him, or one of his loved relatives has died—then a set-point will be adopted that serves to determine and signal the impact of the event on his normal affective state.

Once a set-point related to the sense of self or to one of its dimensions has been adopted, a deviation from it signals how much the object is threatening the sense of self and its integrity; whether the boundaries of the sense of self must be redefined; and whether the person needs to revisit his predictions and expectations concerning himself. *It is precisely this kind of information that the emotion proper conveys to the person. In this sense, emotions are a crucial means through which a person continuously defines and maintains his sense of self*.

Depending on which dimension of the sense of self the person focuses on, and consequently on which set-point is adopted, different emotions arise. A focus on his body, physical wellbeing, or bodily integrity elicits emotions such as fear, anxiety, relief, and disgust; a focus on his cognitive and practical abilities elicits emotions such as pride, curiosity, interest, discouragement, and surprise; a focus on his affective and sentimental life elicits emotions such as love, sadness, grief, and trust; a focus on his moral values elicits emotions such as anger, contempt, guilt, shame, and embarrassment; a focus on his social attitude elicits emotions such as envy, jealousy, hate, gratitude, and admiration.

It must be noted that although several scholars (Snygg and Combs, 1949; Wood et al., 1990; Sedikides, 1992; Tangney, 2005; Abele et al., 2005; Silvia et al., 2006; LeDoux and Brown, 2017; Reeve, 2022) have observed that the experience of emotion is essentially based on an act of focusing one’s attention on one’s own internal state^4^—an act through which the sense of self is brought to the foreground of consciousness—its empirical recognition has not been entirely straightforward or devoid of debate. Initially, several studies, using different manipulations and measures, confirmed that negative affect increases self-focus (Salovey, 1992; Sedikides, 1992; Wood et al., 1990). However, the evidence for positive affect was more controversial: while some studies found that positive affect increases self-focus relative to neutral affect (Salovey, 1992; Silvia and Abele, 2002), others failed to find any relation (Sedikides, 1992) or found that positive affect decreases self-focused attention (Green et al., 2003). The controversy was resolved by the findings of Abele et al. (2005), who showed that positive affect boosts self-focus only if the situation allows it—that is, if the situation is not demanding; if, in contrast, the situation is pressing and demanding, then positive affect leads to a decrease in self-focus.

As noted above, once the conscious emotional experience arises, it promotes further cognitive processing that enables the subject either to restore the aOA’s energy level to its set-point (as usually occurs in the case of negative emotions) or to maintain the changes in that level (as usually occurs in the case of positive emotions). The types of cognitive processes involved can vary. Emotion can modulate:

(i) attention, leading to faster processing of emotional stimuli, making it harder to disengage from them, carrying over its effects to the processing of subsequent stimuli (Anderson, 2005; Fredrickson and Branigan, 2005; Smith et al., 2006; Rowe et al., 2007; Vuilleumier and Brosch, 2009; Yiend, 2010; Brosch and Van Bavel, 2012; Brosch et al., 2013), and selecting the attentional profile most appropriate for the realization of the functional role of the emotion (Mitchell, 2023);
(ii) perception, influencing the most basic perceptual abilities, such as enhancing contrast sensitivity (Phelps et al., 2006), determining whether one perceives global or local stimuli (Gasper and Clore, 2002; Huntsinger et al., 2010), and affecting the perception of spatial features such as slant and distance (Stefanucci et al., 2008; Riener et al., 2011), as well as height and size (Teachman et al., 2008; Stefanucci and Proffitt, 2009; Stefanucci and Storbeck, 2009);
(iii) memory, both by privileging the processing of emotional stimuli at multiple stages—such as rehearsal, consolidation, and retrieval—and in multiple memory systems—such as working memory, and long-term memory—and by impairing the memory processing of neutral stimuli (Levine and Edelstein, 2009; Brosch et al., 2013);
(iv) decision making, guiding choices and judgments (Brosch et al., 2013; Lerner et al., 2015);
(v) learning, promoting the acquisition of new knowledge and forming novel associations between affective states and behavioral responses—the resulting affective traces subsequently influencing behavior without necessarily developing into fully conscious emotional experiences—and allowing individuals to anticipate emotional consequences and act in ways that foster the emotions they find preferable (Baumeister et al., 2007).

## 6 Relation of the AME theory of consciousness to other theories of consciousness

In this article, I have discussed almost exclusively how the AME theory of consciousness explains the emergence of emotions and the mechanisms that enable us to experience them consciously. This theory is not the only one addressing this issue. In this section, I compare it with some of the theories that come closest to it, in order to highlight the similarities and differences between them, thereby allowing for a clearer understanding not only of how the AME theory of consciousness fits within the landscape of current theoretical and scientific research, but also of the possible explanations that it can provide and that other theories cannot.

According to Damasio (1999, 2010), emotions are complex, stereotyped patterns of response, which contribute directly to the regulatory processes of homeostasis. He distinguishes between: background emotions (such as malaise, calm, or tension), which have a low level of specificity and primarily target the internal milieu; primary emotions (happiness, sadness, fear, anger, surprise, and disgust), which are evolutionarily ingrained, largely automatic, have a higher level of specificity, and focus their responses on the musculoskeletal and visceral systems; and secondary emotions (such as embarrassment, jealousy, and pride), which involve learned and culturally mediated evaluations. In his account, emotions precede consciousness and do not depend on it: “Emotion was probably set in evolution before the dawn of consciousness and surfaces in each of us as a result of inducers we often do not recognize consciously” (Damasio, 1999, p. 37). Consciousness of emotions arises only subsequently, when they are represented in the mind as “feelings of emotions” and “feelings of feelings.” Damasio defines feelings of emotions as “composite perceptions of what happens in our body and mind when we are emoting (…) feelings are images of actions rather than actions themselves” (Damasio, 2010, pp. 109–110). Importantly, feelings of emotion result from—and are “complex musical variations on”—more primitive forms of feeling, namely “primordial feelings” (Damasio, 2010, p. 21; see also pp. 110 and 185). Primordial feelings are “felt images of the body” (Damasio, 2010, p. 188) and possess “a definite quality, a valence, somewhere along the pleasure-to-pain range” (Damasio, 2010, p. 185). They provide “a direct experience of one’s own living body, wordless, unadorned, and connected to nothing else but sheer existence” (Damasio, 2010, p. 21), and represent an “immediate manifestation of sentience” (Damasio, 2010, p. 22).

Damasio’s theory and the AME theory of consciousness share important aspects: (i) both assign a regulatory role to emotion, which serves to maintain or restore the organism’s equilibrium; (ii) for both, emotions contribute to the construction of the self; (iii) both reject Cartesian dualism and endorse a unitary, processual conception of the mind, in which body, brain, and consciousness are different aspects of a single dynamic system.

However, they diverge markedly due to the clear separation that Damasio establishes between emotions and feelings/consciousness. For Damasio, emotions precede consciousness and can exist independently of it, whereas in the AME theory of consciousness emotions are intrinsically bound—via attentional activity—to the very construction of conscious experience.

In my view, this sharp separation between emotions and feelings/consciousness significantly hinders Damasio’s ability to develop a theory of consciousness capable of explaining its phenomenological aspect. Indeed, he does not explain it at all; rather, he introduces it surreptitiously when, accounting for its emergence, he claims that: “Whenever brains begin to generate primordial feelings (…) organisms acquire an early form of sentience” (Damasio, 2010, p. 26, italics mine). But how was this “sentience” attained? Where does the human capacity for being “sentient” come from? Damasio does not explain it. He merely states that primordial feelings are something that is “felt” or has “a definite quality,” leaving unexplained what makes these feelings feel the way they do for an organism. Had he realized that emotions (as well as other forms of experience) and feelings/consciousness share a common basis and origin, he would very likely have noticed that they could be traced back to a single generative mechanism—which, in the AME theory of consciousness, is accounted for as the modulation of the aOA’s energy level.

According to Seth’s (2013) and Seth and Friston’s (2016) theory, subjective feeling states (emotions) arise from actively inferred generative (predictive) models of the causes of interoceptive afferents. Their theory generalizes appraisal theories of emotion, which account for the emergence of emotions in terms of cognitions and beliefs about the causes of physiological changes. In their view, the most likely candidates for—or correlates of—conscious emotional experience are “deep expectations at higher levels of the neuronal hierarchy (…) largely because their predictions are domain-general and can therefore be articulated” (Seth and Friston, 2016, p. 5).

Seth and Friston’s theory and the AME theory of consciousness share some important aspects. Both reject the traditional stimulus–response conception of emotion. They conceive of emotional processes as active, constructive, and regulatory rather than as mere reactive outcomes of physiological arousal. In Seth and Friston’s framework, emotions arise from the brain’s ongoing attempts to minimize prediction errors between descending interoceptive predictions and ascending interoceptive signals. This process allows the brain to maintain homeostatic and allostatic balance through active inference. Similarly, in the AME theory of consciousness, emotions are understood as functional modulations of the organism’s internal dynamics that aim to restore or maintain equilibrium in the energy level of the aOA.

A second point of convergence lies in the adoption of an internal set-point. In Seth and Friston’s formulation, descending predictions provide a homeostatic set-point against which primary (interoceptive) afferents can be compared. The resulting prediction error then drives sympathetic or parasympathetic effector systems to ensure homeostasis or allostasis. In the AME theory of consciousness, the set-point of the energy level of the aOA serves to define the hedonic dimension of conscious experience and ultimately to ensure the wellbeing of the individual. Thus, although the implementational details differ (biophysical predictions vs. attentional energy dynamics), both frameworks posit internal reference states that organize correction, anticipation, and the assignment of significance to bodily and experiential changes.

Despite these shared aspects, the two theories diverge on several decisive points. First, the status of attention sharply distinguishes the two theories. In Seth and Friston’s theory, attention is conceived as precision-weighting—that is, as a modulation of the gain of prediction errors, determining which signals (bottom-up vs. top-down) are afforded higher confidence in updating the generative model. Attention thus has a secondary, modulatory function. In contrast, in the AME theory of consciousness, attention is the core activity that constitutes consciousness and emotion. Emotions are variations in the energy level of the aOA; they are not inferred or computed but experienced as modulations of the attentional field.

Second, the two theories diverge fundamentally in their account of conscious experience. Seth and Friston conceive consciousness as the outcome of internal, hierarchically structured models that the brain uses to stand in for and to explain the causes of sensory signals. These models operate within a broadly representationalist and inferential framework that does not ground experience it in the lived, first-person structure of consciousness. Unfortunately, the mere existence of internal models of the external world cannot explain the emergence of subjective consciousness, since it presupposes the very existence of the phenomenological subject rather than accounting for how it forms and develops (Di Paolo et al., 2022; Marchetti, 2022; Pae, 2025). By contrast, in the AME theory of consciousness, consciousness is not the product of a representational model but a dynamic attentional act: a self-organizing field of activity that unifies the organism’s internal state with its relation to the object. Crucially, this account allows one to explain the pre-reflective givenness of experience: attentional working creates consciousness as it is lived from the first-person perspective, making experience—whether emotional or otherwise—immediate, intrinsic, and not merely inferred or represented.

Third, the two theories also diverge in the function they assign to emotion. For Seth and Friston, emotion primarily serves homeostatic and allostatic functions—regulating physiological variables and ensuring adaptive control. In the AME theory of consciousness, emotion has a broader organizing function: it structures conscious experience and the relationship between self and world, thereby shaping both cognition and identity. It is not merely a mechanism for bodily regulation but a fundamental mode of experiencing and constituting reality.

In conclusion, the features of Seth and Friston’s theory, while providing a precise biological and computational account of how predictive mechanisms contribute to certain aspects of interoception and emotion, render it ill-suited to explain the subjective, phenomenological dimension of consciousness in general, as well as the manner in which emotional states, in particular, are experienced and constituted within conscious awareness.

Remaining within the context of predictive-inferential frameworks, I cannot help but mention the work of Solms (2019), if only because of its very close proximity to the AME theory of consciousness and the explicit way in which he presents his account. According to Solms, “consciousness is fundamentally affective” (Solms, 2019, p. 6), in the sense that affect (the technical term for feeling) is the elemental form of consciousness, grounded in brainstem-based homeostatic mechanisms formalized in terms of free-energy minimization. Affect enables complex organisms to register, regulate and prioritize deviations from homeostatic settling points in unpredicted contexts.

A first important common point between Solms’ theory and the AME theory of consciousness is the recognition that the hedonic dimension of consciousness arises from deviations from a homeostatic set-point: in his view, the demand—or “drive”—made upon the mind for work in consequence of a deviation from a homeostatic set-point *is* the feeling (Solms, 2019, p. 7).

A second common point is the definition of pleasure and unpleasure as deviations toward and from the homeostatic set-point, respectively: “Affective qualia are accordingly claimed to work like this: deviation away from a homeostatic settling point—increasing uncertainty—is felt as unpleasure, and returning toward it—decreasing uncertainty—is felt as pleasure” (Solms, 2019, p. 7).

Finally, the two theories share the view that a complex system such as the human organism, being composed of various subsystems, requires that the various homeostatic demands arising from each subsystem are orchestrated and managed at the level of the organism as a whole. Solms generally identifies the nervous system as the organ responsible for performing this orchestration—“Nervous systems are therefore meta-systems, performing meta-homeostatic functions on behalf of the entire body. Homeostatic regulation of the organism as a whole is delegated, as it were, to the nervous system” (Solms, 2019, p. 10)—while the AME theory of consciousness specifically identifies it with the organ of attention.

Most likely, this first difference between the two theories leads Solms to overlook the possibility that this very meta-system can also be charged with the task of generating the other dimensions of conscious experience—qualitative, quantitative, spatial, and temporal—on top of the hedonic one.

Another remarkable difference, due to the fact that Solms adopts the predictive-inferential framework, lies in the reduction of the function of consciousness to detecting uncertainty: “Consciousness adaptively determines which uncertainties must be felt (i.e., prioritized) in any given context. In short, consciousness is felt uncertainty” (Solms, 2019, p. 7). It is certainly undeniable that consciousness also serves this function, but restricting consciousness to only this role seems unwarranted to me: in fact, there are many occasions in our lives in which we seek certainty, stability, and calm.

A final difference is that, whereas Solms locates the generative mechanisms of consciousness in specific subcortical nuclei—“we must conclude that consciousness is generated in the upper brainstem” (Solms, 2019, p. 6)—the AME theory of consciousness identifies the generative mechanisms of consciousness with the organ of attention, which, being a distributed functional system performing multiple functions, most likely involves both subcortical and cortical brain areas (on this point, see Marchetti, 2006).

I conclude this section by drawing on some of the results that the comparison between the AME theory of consciousness and alternative accounts has revealed. Specifically, the differences highlighted by this comparison allow for the identification of empirically testable predictions that distinguish the AME theory of consciousness from alternative accounts. Table 2 summarizes some of these predictions, the kinds of evidence that would support them, and the kinds of findings that—by supporting the predictions of alternative theories—would instead disconfirm the AME theory of consciousness. Table 2 also includes an empirical prediction that differentiates the AME theory of consciousness from the theory of LeDoux and Brown (2017).

**Table 2:** Predictions of the AME theory of consciousness, contrasts with alternative theories, and the relevant supporting and falsifying evidence.

Theoretical issue	Predictions of the AME theory of consciousness	Prediction of alternative theories	Evidence supporting the AME theory of consciousness	Evidence falsifying the predictions of the AME theory of consciousness and supporting those of alternative theories
Role of attention vs. interoception in emotional consciousness	Conscious emotional states require attention to be allocated to affective contents; without sufficient attentional engagement, emotional states do not become phenomenally conscious, regardless of interoceptive processing	Conscious emotional states arise from predictive inferences about interoceptive signals; attention modulates precision but is not constitutive (Seth and Friston, 2016).	Experiments showing that targeted manipulations of attention (with interoceptive variables held constant) systematically alter the phenomenal aspect of conscious emotion, while interoceptive manipulations without attentional changes have null or weaker effects.	Evidence that interoceptive perturbations reliably produce conscious emotional states even when attention is fully absorbed by unrelated tasks.
Neural locus of the generative mechanisms of consciousness	Consciousness emerges from the modulation of the energy of the attentional organ, which is not localized in a single subcortical brain structure but most likely involves both subcortical and cortical brain areas. The brainstem is necessary for the level of arousal but not sufficient for rich conscious phenomenology.	Consciousness originates primarily in the upper brainstem; cortex contributes content but not the core phenomenology (Solms, 2019).	Experimental manipulations (e.g., TMS) of cortical attentional networks, altering experience quality under stable arousal.	Evidence showing rich phenomenology—i.e., differentiated and structured conscious experience—generated solely by the brainstem, without cortical involvement.
Relation between attention and consciousness	There cannot be consciousness in the complete absence of attention; different forms and levels of attention generate different forms and levels of consciousness.	Consciousness (as affective feeling) can exist independently of attention; attention mainly selects and structures contents already conscious (Solms, 2019).	Experiments showing that when any form of attentional processing is completely prevented, conscious experience is abolished rather than merely degraded.	Robust evidence of fully conscious experiences occurring in the absence of any form of attentional processing, including bottom-up and diffused attention.
Conscious vs. unconscious emotions	Emotions can be fully conscious, partially conscious, or entirely unconscious, depending on the level of attentional processing.	Emotions can never be unconscious; only defensive survival circuits operate unconsciously. Emotional feelings require higher-order cortical representations (LeDoux and Brown, 2017).	Experimental paradigms (e.g., dual-task) in which attention is partially or fully absorbed, reducing or eliminating conscious feeling while leaving emotional biases, preferences, or action tendencies intact.	Evidence showing absence of emotional effects without conscious feeling: if subjects who report no emotion also show no behavioral, physiological, or cognitive effects, this would indicate that emotions require consciousness.

## 7 Conclusion

In this article, I have aimed to explain how emotions arise and operate within the framework of the AME theory of consciousness. According to this theory, conscious experience is primarily determined by the attentional activity we perform, which modulates the energy level in the area of the neural organ of attention (aOA) supporting that specific activity. It is precisely this modulation that generates the phenomenal aspect of consciousness. This modulation accounts for the five main dimensions of consciousness—qualitative, quantitative, hedonic, temporal, and spatial—and, consequently, for the specific phenomenology of each conscious state.

Each of the five dimensions of conscious experience contributes, in its own way, to shaping the key features of the sense of self: the sense of being an entity differentiated from others, the point of view or perspective, the feeling of continuity, and the sense of unity.

The AME theory of consciousness offers a novel framework for understanding emotions as both products and regulators of the dynamic relationship between consciousness, the Self, and the sense of self. By explaining emotions as emerging from attentional modulation processes—which affect the energy level of the area of the aOA—the AME theory of consciousness links the phenomenology of emotional experience to its functional and informational foundations. From this perspective, emotions are not merely transient affective states but fundamental operations through which the individual monitors, defines, and maintains his sense of self.

Emotions emerge from the interaction of three main components—core affect, cognitive appraisal processes, and physiological and behavioral manifestations—whose interplay develops through successive cycles of conscious and unconscious processing (the latter occurring within the Self). They arise when the focus of attention shifts from the object that initially elicits the affective reaction to the sense of self. This shift activates a new aOA and a corresponding set-point related to a specific dimension of the sense of self (bodily, cognitive, affective, moral, social, or other). Deviations from this set-point constitute the core of the conscious experience of an emotion proper.

Through this shift from the object to the sense of self, emotions provide the individual with essential information about the state of his internal equilibrium, the boundaries of his sense of self, and the adequacy of his self-representations and expectations. In doing so, they not only guide adaptive behavior but also contribute to the continuous construction and renewal of his sense of self.

This view invites a reconsideration of the traditional distinctions between cognition and emotion, and between the conscious and the unconscious, showing that these domains are deeply intertwined manifestations of the same attentional dynamics, with emotions occupying a central role in mediating between them.

Further research may refine the AME theory of consciousness by exploring, both theoretically and empirically, how the modulation parameters of the aOA—its amplitude, polarity, direction, and speed—correspond to specific emotional qualities, and how attentional cycles coordinate the alternation between conscious and unconscious processing during emotional episodes. Such investigations could not only illuminate the mechanisms underlying emotional awareness but also deepen our understanding of consciousness itself, conceived as a process of continuous self-regulation through attention.

## Data Availability

The original contributions presented in the study are included in the article/supplementary material, further inquiries can be directed to the corresponding author.

## Footnotes

1 See also Cabanac (1979), p. 21: “Pleasure is actually observable only in transition, when the stimulus aids the subject to return to normothermia. As soon as the subject returns to normothermia (…), all stimuli lose their strong pleasure component and tend to be indifferent or unpleasant.”

2 I partly owe this perspective to Cabanac (2002), who claims that an emotion is any mental experience characterized by both high intensity and high hedonicity. However, I diverge from Cabanac when he specifies that a mental experience of either low intensity or low hedonicity does not qualify as an emotion. I find this view somewhat restrictive, as it excludes emotions typically regarded as low in intensity—such as sadness, boredom, discontent, and melancholy—and emotions regarded as low in hedonicity—such as apathy—that are persistent and difficult to resolve or extinguish.

3 At first glance, this notion may seem quite extreme: one could indeed argue that certain events—such as seeing another person bleeding—inevitably elicit some emotion in us, or that witnessing someone crying or laughing induces us to feel the same emotion. However, this is not always the case. Professionals such as doctors can, and often must, perform their work without feeling any emotion toward the blood or wounds they see. Likewise, seeing someone sarcastically laughing at the misfortunes of others does not always automatically make us laugh.

4 This does not exclude, however, the possibility of self-focused attention occurring without subjective emotional experience (Silvia et al., 2006).
